# Computer Mediated Automatic Detection of Pain-Related Behavior: Prospect, Progress, Perils

**DOI:** 10.3389/fpain.2021.788606

**Published:** 2021-12-13

**Authors:** Kenneth M. Prkachin, Zakia Hammal

**Affiliations:** ^1^Department of Psychology, University of Northern British Columbia, Prince George, BC, Canada; ^2^The Robotics Institute, Carnegie Mellon University, Pittsburgh, PA, United States

**Keywords:** pain, measurement, facial expression, automation, assessment

## Abstract

Pain is often characterized as a fundamentally subjective phenomenon; however, all pain assessment reduces the experience to observables, with strengths and limitations. Most evidence about pain derives from observations of pain-related behavior. There has been considerable progress in articulating the properties of behavioral indices of pain; especially, but not exclusively those based on facial expression. An abundant literature shows that a limited subset of facial actions, with homologs in several non-human species, encode pain intensity across the lifespan. Unfortunately, acquiring such measures remains prohibitively impractical in many settings because it requires trained human observers and is laborious. The advent of the field of affective computing, which applies computer vision and machine learning (CVML) techniques to the recognition of behavior, raised the prospect that advanced technology might overcome some of the constraints limiting behavioral pain assessment in clinical and research settings. Studies have shown that it is indeed possible, through CVML, to develop systems that track facial expressions of pain. There has since been an explosion of research testing models for automated pain assessment. More recently, researchers have explored the feasibility of multimodal measurement of pain-related behaviors. Commercial products that purport to enable automatic, real-time measurement of pain expression have also appeared. Though progress has been made, this field remains in its infancy and there is risk of overpromising on what can be delivered. Insufficient adherence to conventional principles for developing valid measures and drawing appropriate generalizations to identifiable populations could lead to scientifically dubious and clinically risky claims. There is a particular need for the development of databases containing samples from various settings in which pain may or may not occur, meticulously annotated according to standards that would permit sharing, subject to international privacy standards. Researchers and users need to be sensitive to the limitations of the technology (for e.g., the potential reification of biases that are irrelevant to the assessment of pain) and its potentially problematic social implications.

## Introduction

The International Association for the Study of Pain's recent revision to the definition of pain [“an unpleasant sensory and emotional experience associated with, or resembling that associated with, actual or potential tissue damage;” (1)] added several contextualizing notes. First, pain is “always a personal experience, influenced…by personal, psychological, and social factors.” Second, “a person's report of an experience as pain should be respected.” Lastly, verbal description is only one of several behaviors to express pain.” The first and second recognize that the experience of pain is subjective and falls into the category of phenomena we call “feelings.” The second addresses the common temptation, when a phenomenon is subjective, to be skeptical about its reality or its potential to be interrogated scientifically. The third recognizes that evidence about pain exists in various types of behavior. While we can acknowledge that there is much in the experience of pain that is unique and individual, if we are interested in advancing understanding of pain, either from a purely scientific point of view or for utilitarian purposes of management and control, then we must achieve some consensus on the evidence we use to infer its presence and properties.

The experience of pain cannot be directly measured. Instead, there are two general categories of pain indicators. One consists of changes in the body, especially but not limited to the central nervous system, that are believed to mark and quantify pain and that can be measured more-or-less directly by some form of instrumentation. The other consists of behavior. The vast majority of pain indicators, including verbal descriptions, fall into this category. Other behavioral pain indicators include instrumental acts, such as withdrawal or avoidance and expressive acts, such as vocalizations or grimacing.

In recent years advances in technology, accompanied by expanding analytic tools in the area of computer vision and machine learning (CVML), have been applied to some behavioral pain indicators in efforts to improve on them for both scientific and practical reasons. Until recently, most progress has been made toward automatic assessment of facial expression of pain (2, 3). Although in everyday pain experience we encounter associations between body movement and pain, the communicative functions of body movements in relation to pain have been fairly unexplored in automatic pain assessment. Notable exceptions are to be found in the work of Aung et al. [(4), see also Egede et al. (5)] who found association between pain and certain bodily protective behaviors, such as guarding/stiffness and bracing/support.

In this article, we describe the advent of such approaches, as they relate to facial expressions of pain, beginning with the behavioral roots that gave rise to them. We articulate the prospects foreseen for such approaches, then describe early progress in the form of “demonstrations of concept.” We then go on to summarize key developments and address emergent applications of the work, including the development of commercial products. In the course of this narrative, we highlight emergent problems that, we believe, should qualify enthusiasm about the field.

## Verbal Assessment of Pain

While it is possible to gain insight about a subjective process, that insight often comes indirectly—by operationalizing it in the form of a measure. In the field of pain, operationalized verbal reports have become a standard—indeed it is common to see verbal report referred to as the “gold standard.” Verbal reports of pain can be obtained about different dimensions but pain intensity is overwhelmingly the most frequently assessed. The widely used visual analog scale (VAS), in which the respondent marks a spot on a line of finite length to characterize their pain, is a variation on verbal report. In clinical and population-based studies, verbal descriptor or VAS scales are commonly used to characterize certain pain states or as outcome measures in studies of interventions. The 0–10 numeric rating scale was advocated for and implemented widely in health-care settings as a fifth vital sign.

### Limitations of Verbal Assessment of Pain

The fact that verbal report techniques are used ubiquitously is a testament to their utility. However, concerns about their potential shortcomings are common. One concern is epistemological, reflecting an underlying belief that scientific inquiry should be based in measurements of things that are objectively observable. But there are others. For one, verbal reports bear an uncertain relation to the underlying experience. They can be shown to behave in a way that should coarsely correspond to an underlying pain state, such as when people use lower numbers or words reflecting lesser pain to describe their pain after being administered a known analgesic. However, when a patient with low back pain who initially gave a rating of 8 to their pain now gives a rating of 4 after a rehabilitation program does that mean they are in half as much pain? In the historical debates about pain measurement, this issue was at the center of several attempts to develop psychophysical techniques with ratio-scale properties (6–8).

Even if it can be shown that verbal ratings vary according to expectations in experimental and clinical studies, it is not possible to be certain that all individuals use the scales in the same way. Some people are more sensitive to variations in the experience and more precise reporters than others. Williams et al. (9), for example, reported a lack of concordance between patients and consistency within patients in their use of visual analog and numeric rating scales as they actively interpreted the meaning of their experiences.

Often, variations in the operationalization reveal inconsistencies in the characterization. When different techniques are used to assess the painfulness of the same level of nociceptive stimulation in experimental studies, or the same patient at the same time in clinical studies, the evaluations are often incommensurate. For example, in one of our recent studies, participants were asked first to rate cold pressor pain using a VAS. Then, at the end of the study, they were asked to rate the maximum pain using the pain intensity rating (PIR) of the McGill Pain Questionnaire. Participants who gave the maximum pain rating according to the VAS—a rating corresponding to “worst pain imaginable” frequently gave a PIR rating implying pain of considerably lower intensity.

One of the most well-known features of verbal reports is their extraordinary malleability. This property has been known for a long time, featuring in Beecher's (10) classic *Measurement of Subjective Responses* in the form, among other things, of the placebo effect. Craig's early studies of the social modeling effect [e.g., (11)], showed that exposure to tolerant or intolerant social models could make participants rate electric shocks less or more painful, respectively. Such malleability may, of course, simply exemplify that pain is an extremely plastic phenomenon. On the other hand, recognizing that verbal report is under exquisite control of the perceiver raises concern whether what is being measured is instead the response to personal or social expectations embedded in the conditions of observation such as expectancy effects or demand characteristics, independent of any true effect on the pain experience itself. One example of the concern arose in studies of hypnosis that made use of the “hidden observer” technique (12). Participants under hypnosis were given suggestions that they would experience an analgesic state. They then rated the painfulness of cold in the cold-pressor test. Participants were also told that under hypnosis they would have access to the experience of their hidden observer—a part of them that would experience the pain as it was—and that they were to give the ratings of the hidden observer after they rated their own pain under hypnotic analgesia. The studies showed a dissociation between the ratings of the hypnotized subject and the same subject's hidden observer Spanos and Hewitt (13), however found that the hidden observer's ratings could be easily diverted by manipulations of what the participant expected that the researcher expected.

Similarly, self-presentation biases are likely to come into play and distort controlled verbal reports in a species as socially responsive as humans. A common self-presentation bias in the pain context is stoicism. When self-report is the criterion, studies (both clinical and experimental) routinely find, for example, that men report lower pain than women (14). It is, of course, possible that this reflects a true difference in pain sensitivity between the sexes, but there is an obvious socialization difference in which masculinity is equated with enduring pain that can also account for the difference.

A final shortcoming of verbal report in studies of pain is that there are important instances in which verbal reports cannot be obtained because the respondent is incapable of using words to describe their pain (for example, preverbal infants, people with profound verbal communication impairments and non-human animals), or people who, though capable of communicating verbally, are impaired in the ability to communicate reliably about pain (such as in types of dementia).

## Physiological Assessment of Pain

There is a substantial history of search for alternatives to self-report. A diversity of physiological measures has been promoted over the years, including measures of autonomic responses such as electrodermal activity (15), oxygen saturation (16), heart-rate variability (17), and evoked potentials (18). With the advent of neuroimaging procedures measures of regional cerebral bloodflow have become ubiquitous in pain studies. Some have been promoted as true “central registers” of the pain experience, but none are widely recognized as such (19).

### Limitations of Physiological Assessment of Pain

A physiological measure of pain has been a kind of “holy grail” among some researchers and clinicians. Physiological variables such as those noted are routinely deployed in both basic and clinical studies but have not achieved consensual status as measures of pain outcomes. Some, such as electrodermal activity or heart-rate variability, serve as indices of processes that are affected by pain, such as autonomic arousal. As measures of pain, they are sometimes overly responsive and therefore poorly discriminating of variations in pain states, sometimes insufficiently responsive and therefore also poorly discriminating, and sometimes covary with other affect states with which pain is correlated, such as fear. Neuroimaging procedures have identified various brain regions in which activation varies in accordance with other evidence of pain; however, they are distributed across networks in a manner that does not lend itself to simple interpretation as pain indicators. Most physiological assessment techniques are at least modestly invasive, involving special instrumentation and sometimes highly specialized laboratory environments and therefore do not lend themselves to study in ecologically normative conditions.

## Pain Assessment Based on Fine-Grained Facial Observation

The insight that behavior is fundamental to the understanding of pain gained currency with the development of behavioral approaches to pain management. As Fordyce (20) observed, a person has to do something for it to be known that they are in pain. The early behavioral approach was based in the learning theory of the day but did not make nuanced distinctions about the properties of pain-related behaviors that varied by topography.

The model brought an emphasis on observation and precise definition and assessment of behaviors that, curiously, dovetailed with the concerns of students of emotion.

The study of emotion had venerable roots in the work of Charles Darwin. In *The expression of the emotions in man and animals*, Darwin (21) argued that emotions are phylogenetically shared with other species. He described how various affective states, including pain, are represented in specific behavioral topographies, especially but not exclusively facial expression.

Interest in the role of the face in communication of affect revived in the late 1960's, reflecting in part the influence of studies supporting the idea that facial expressions of certain emotions are universal across human cultures (22). Subsequent refinements in methods for studying facial expressions laid the foundation for examining their role in communicating information about pain.

In 1978, Ekman and Friesen published the Facial Action Coding System (FACS). This is a system for deconstructing any facial movement into its constituent actions based on the changes that appear when an individual muscle or combination of muscles are activated. Observers trained to FACS proficiency then view facial expressions and describe their constituent actions in terms of 44 action units (AUs) or action descriptors (ADs). Most AUs can be described in terms of their intensity. Intensity coding for most AUs is on a 6-point A—E scale, where a code of A is assigned to a trace of an action, B to an action that meets minimum requirements for the action, E to an action that is as strong as it could be, and codes in between refer to gradations between meeting the minimum requirements and maximum intensity (note that, in quantitative analyses the alpha codes are transformed to numbers between 1 and 5; if the action has not occurred a code of 0 is assigned as default).

The system is thus anatomically based, atheoretical, and relatively objective (“relatively” because inferences are still involved; for example, when rating intensity). It is manualized such that, with intensive study, an observer can learn the system within about 100 h. Data quality when performed by observers who have established proficiency in the system by passing a proficiency test, is generally sufficient to meet conventional reliability standards and the system is generally considered to be the “gold standard” for assessing facial action.

The FACS has been applied extensively in studies to characterize the appearance of the face when a person is in pain. A systematic review of 37 studies (23) reported that, for both experimental and clinical pain, a subset of facial actions reliably discriminates between pain and no-pain conditions. These are: brow lowering (FACS AU 4), orbit tightening (AUs 6 or 7), levator tightening (AUs 9 or 10), and mouth opening (AUs 25, 26, or 27). Eyelid closing (AU 43) also consistently discriminates between pain and no pain in studies of clinical pain. The same actions discriminated pain from no pain independent of the participants' cognitive status (impaired vs. unimpaired).

Systems resembling FACS have been developed for studies of pain in children. The two systems that have been applied most widely are the Neonatal Facial Coding System [NFCS; (24)] and the Child Facial Coding System [CFCS; (25)]. Rather than being defined by the underlying facial musculature of the constituent actions, NFCS and CFCS codes are based on appearance changes. In both neonates and young children, the codes that have been found most consistently to discriminate pain from no pain conditions are homologous to the codes that distinguish pain from no pain conditions in adults, including seniors; namely, brow bulge (NFCS)/brow lower (CFCS), eye squeeze (both systems), nasolabial furrow /nose wrinkle (NFCS), nasolabial furrow, upper lip raiser (CFCS) (26). Various other facial actions have been associated with pain in neonates and children. Nevertheless, the smaller “core” subset appears with remarkable consistency across types of pain and the human life-span, including among the aged. There is also a noteworthy similarity with the facial actions reported to be associated with pain in non-human animals that have been studied to date [e.g., (27)].

### Limitations of Fine-Grained Facial Observation of Pain

Somewhat remarkably, despite the substantial scientific literature documenting the properties of facial expressions of pain, the work has had little application in basic science or clinical studies of pain. The simple reason for this is that objective description of facial action by FACS or similar systems is burdensome. FACS is implemented by human observers who require training to render assessments that are sufficiently reliable for scientific purposes. Implementing FACS in scientific or clinical studies cannot be done practically in real-time because coding requires multiple observations of behavioral samples to identify the separate actions of separate muscle groups. Ordinarily it requires slow-motion and stop-action to settle on a final set of codes. This makes the coding process lengthy–a final code from a sample of behavior is typically estimated to require a coding time: real time ratio of around 100:1. Conducting studies with requisite numbers of participants and observations quickly becomes arduous and, for human observers, oppressive. Realistic application in clinical settings is impractical. Although some work has aimed at reducing training and coding time by focusing on only facial actions that have been empirically associated with pain (28), even modified procedures are problematically time-consuming. Further, the measurement rendered by human observers is insufficiently granular and continuous to render certain kinds of information that could provide the insights into pain processing that the face may be capable of; for example, temporal information about the onset and decay of certain facial actions that may be informative about such issues as the relative reflexivity or conscious modulation of the sufferer.

For these reasons, since the inception of fine-grained systems for measuring facial action, there has been an underlying question whether advances in information technology could render a technique as reliable and valid as facial coding by trained observers that would reduce the burden of observation, that would not be subject to human observers' susceptibility to fatigue and error, that might be more sensitive and better able to represent dynamic changes. Development of the field of affective computing appeared to address this prospect.

## Toward Automated Assessment of Pain From Non-verbal Behavior

Affective computing has been defined as “computing that relates to, arises from, and deliberately influences emotion” (29, 30). It subsumes a wide range of topics and applications, one of which is the measurement and modeling of affective processes. Affective processes like pain have behavioral markers, including but not limited to facial expressions, that can be captured and stored by technology. Decoding their messages is a kind of pattern recognition. Advances in computer and data science enabled by the development of neural nets and machine learning, which had proved to be successful modeling pattern recognition, appeared to offer a technological solution to the burden associated with decoding facial expressions. Further potential benefits, such as rapid processing and the ability to render more precise information about movement dynamics than can be effectively obtained from human observers, appeared possible.

Some of the earliest demonstrations of the feasibility of such automated analysis of facial expression appeared in work by Bartlett et al. (31) and Cohn et al. (32). Bartlett et al. obtained images of FACS upper-face AUs varying in intensity from 20 people. Processed by a two-layer neural network, a hybrid classification system combining holistic spatial analysis, facial feature measurement, and analysis of motion flow fields was able to correctly classify 92% of the six facial actions [of which three (AUs 4, 6, 7) had been implicated in studies of pain], outperformed naïve human judges, and approximated the performance of human experts. Cohn et al. used video frames of 15 FACS AUs or AU combinations as training stimuli. After alignment, facial landmarks were marked and then automatically tracked using an algorithm to estimate optical flow across images. minant function analysis produced 92% or higher agreement with the classifications of a human coder in a training set and between 81 and 91% (depending on facial region) in a cross-validation set. These studies strongly suggested that advances in computer vision methods combined with advanced statistical analysis could, in principle, make automated analysis of facial expression possible.

The advent of techniques to automatically measure facial expressions naturally stimulated interest in extending the technology to the measurement of facial expressions of pain. Effective automated assessment held promise to overcome barriers to more widespread scientific and practical applications of facial expression measurement. In principle, it could reduce or eliminate the need for human coders thereby managing the problem of observer burden. Once tested sufficiently and validated, an automated system could potentially be more reliable than measurement by human observation because it could reduce variability and human error. Early work on automating measurement of human emotional expressions began to reveal properties of facial action that had been impractical to study. For example, using an automated facial analysis technique, Ambadar et al. (33) showed that different categories of smiling (polite, amused, embarrassed) differed in terms of velocity, duration, and association with head movements. From a scientific perspective, the prospect of an automated system opened the tantalizing possibility of measuring momentary dynamic changes in pain-related facial expressions to draw similar inferences about its meaning and underlying determinants. From a practical perspective, an automatic, objective, reliable, and efficient assay of the occurrence and intensity of pain could improve clinical pain assessment, allowing health-care personnel to provide better treatment to patients, with little to no increase in cost (2). It could also support pharmaceutical therapies by providing an objective quantitative tool for evaluating the efficacy of current and new analgesics and serve as an objective complement to self-reported pain measures in clinical trials of drug or device interventions to reduce pain.

### Methodological Foundations

To learn the association between pain occurrence or intensity and facial behavior, recordings of participants responding to painful conditions are needed in order to train and test classifiers. Samples of sufficient size to estimate training parameters and perform validation analyses are necessary. The number of participants should be motivated by two factors. One is the number needed to achieve saturation in the performance of the predictive models (i.e., automatic classifiers). The other is the number needed to enable sufficient power in the statistical models for quantifying the contribution of the used variables in the predictive models. For instance, in prior work on a related problem (training automated classifiers for facial action units), it was found that automatic classifier performance saturates at about 60 participants in the training set (34). With 25 participants in the UNBC-McMaster Shoulder Pain Expression Archive Database, the number of available participants is far lower than that minimum number needed. Additionally, independent criteria for establishing the absence, presence, or intensity of pain (i.e., “ground truth”) must be present. Although they have not been as widely tested in pain studies, ground truth in automated pain assessment has mostly been derived from annotations by expert observers (using FACS or a variant of FACS) of video recordings of facial expression of pain. However, there must be sampling in conditions in which it is reasonable to assume that pain has occurred (such as during a clinical test, or during exposure to artificially induced painful conditions, such as noxious heat), and in conditions when pain is unlikely. As an alternative or supplement, judgment studies can be performed in which observers (who might vary in expertise) rate recordings on an appropriate scale of pain intensity. Another alternative that has only recently come to be explored is the subjective judgments of participants undergoing the potentially painful procedure. Finally, known conditions can serve as ground truth, such as when, in one experimental condition, a participant is exposed to a stimulus known to cause pain and in another, they are not. If ground truth is based on annotations or ratings by human observers, they must also meet criteria for acceptable reliability. Meeting the aforementioned criteria is a challenging task but has been achieved by several groups [(35–39)].

Because of the power requirements of machine learning and classification procedures, there is an issue related to the density and precision of annotations. Analyses are based on the recordings made in the aforementioned clinical or experimental conditions. A behavioral sample can be annotated at the level of the overall sequence using a single observation or a summary, which yields one measure per sequence. Alternatively, depending on the annotation method, it can be annotated at the level of the individual frame. Whereas, annotation at the level of the frame provides considerable amounts of data for training and validation purposes, annotation at the level of the sequence provides but one per participant and condition, with obvious implications for sampling in the pain recording phase of any study. In either case, but more particularly for studies in which annotation is frame-by-frame, at least in data collected to date, the distribution of pain intensities is problematic, with there usually being a much higher number of frames in which annotations suggest no pain than pain, with implications for training models.

In part because of the resources required to meet the forgoing criteria, but also because experimentation with different CVML methods benefits from comparison and calibration against extant work, databases that can be shared for model testing are desirable. The UNBC-McMaster Shoulder Pain Expression Archive Database [(40, 41)] was the first to address this need. The archive contains video recordings of people with shoulder pain taken during active abduction, flexion, internal and external rotation of their affected and unaffected shoulders (41). It comprises 200 video sequences from 25 different participants (66% female). For each sequence, the distribution includes 66 Active Appearance Model (AAM) tracked landmarks (fiducial points around the eyes, eyebrows, and mouth) at the frame level and per-frame and per-video pain score annotations. Expert labeled FACS codes were scored using a 0–5 ranking of the intensity of the facial actions in most cases. Intercoder agreement as calculated by the Ekman–Friesen formula (42) was 0.95. the participants' self-reported pain intensity and an independent observer's ratings of pain intensity (OPI) were annotated at the sequence level. Offline observer ratings were performed on a 6-point Likert-type scale that ranged from 0 (no pain) to 5 (strong pain). To assess inter-observer reliability of the OPI pain ratings, a second rater independently rated 210 randomly selected videos. The Pearson correlation between the observers' OPIs was 0.80, which represents high inter-observer reliability.

Since being made available to qualified researchers, the Pain Archive has been the most widely used dataset for exploring automatic pain assessment from facial expression, accounting for approximately 41% of the literature published in this field according to a 2019 systematic review (3).

A smaller number of studies (43–49) have made use of BioVid (50), a heat pain database. BioVid contains recordings of 87 people exposed to four intensities of experimental heat pain and a no pain baseline. Each intensity (including no pain) was presented 20 times in a random sequence. Each video excerpt has a duration of 5.5 s. Unlike the UNBC-McMaster Shoulder Pain Expression Archive Database, ground truth is based on stimulus intensity, rather than a measure of pain expression.

A third database, EmoPain (4) contains recordings from 22 adults with low back pain. The recordings were taken while the patients engaged in movements resembling common therapeutic tasks for back pain patients. Data streams include audio recordings, 3D motion capture, and electromyographic recordings from the paraspinal muscles in addition to facial expression. Measures available for ground truth include patient pain and anxiety ratings, and offline observer ratings using a joystick method. EmoPain has not yet been publicly released as had been planned.

### Proof-of-Concept Studies

**Figure 1:**
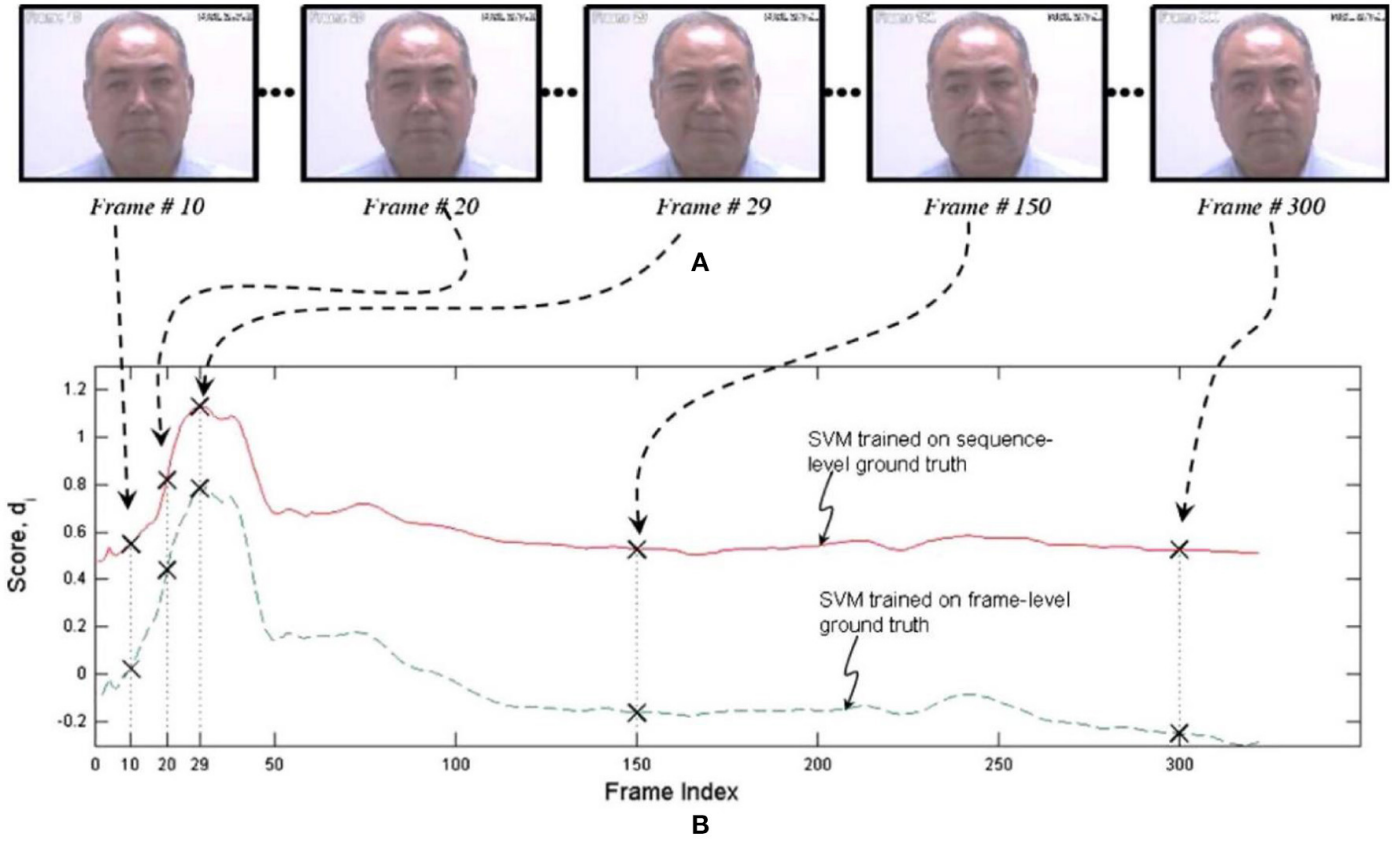
SVM scores for sequence- and frame-level ground truth. The upper pictorial representations **(A)** are the video frames corresponding with the crosses on the respective SVM score plots **(B)** below. Reprinted with permission from Ashraf et al. (51).

One of the earliest efforts to develop an automated system for measuring pain expression appeared in Ashraf et al. (51). The authors employed recordings from the UNBC-McMaster Shoulder Pain Expression Archive Database of shoulder-pain patients described above. They had been quantified at the level of the individual video frame by a FACS-based index of expressive intensity, dubbed the Prkachin Solomon Pain Index [PSPI; (41, 52)], and consisting of the summed scores of AUs that have consistently been associated with pain in observational studies. After transformations to optimize registration of the face, support vector machines (SVMs) were trained to classify full sequences or individual frames as showing pain or no pain. The best combination of representations resulted in hit rates of 77 and 82% for sequence level and frame-level classification, respectively, and false acceptance rates of 44 and 30%, showing that it was possible to obtain reasonable differentiation of pain from no pain states when evaluated with respect to the ground truth of direct facial measurement by trained observers. Unsurprisingly, the more granular frame-level approach provided better performance. Figure 1 displays performance of both approaches for a representative participant.

In another early study of automatic pain detection, Littlewort et al. (36) employed a system for automatic detection of FACS AUs to examine facial changes during exposure to experimental pain produced by immersion of the arm in ice-water and to compare those changes with actions performed when participants pretended to be in pain. Genuine pain was associated with increases in six automatically detected representations of AUs previously associated with cold-pressor pain in studies using human observers. “Faked” pain was associated with 11 automatically coded actions. In a subsequent machine learning phase, automated facial action parameters were processed via a Gaussian SVM in an attempt to discriminate genuine from faked pain. The resultant 2-alternative forced-choice percent correct value of 88% substantially exceeded the performance of naïve human observers at 49%.

Lucey et al. (53), also using the UNBC-McMaster Shoulder Pain Expression Archive Database, applied a system combining Active Appearance Models (AAMs) for tracking face shape and appearance, input to SVM's for pain and AU classification at the level of the individual video frame. Ground truth consisted of expert-coded FACS AUs, including, but not limited to the PSPI. In a test of the system for directly classifying pain (i.e., predicting a PSPI score of >0) the Receiver-Operating-Characteristic (ROC) based A' metric yielded a score of 0.75, indicating performance substantially greater than chance. An indirect classification system, predicting pain from an alternative set of individual FACS AUs that excluded two components of the PSPI and included AU12, performed slightly better, achieving an A' score of 0.77, relative to 0.78 for the PSPI. Building upon those results, Lucey et al. (54) again used a combination of AAM/SVM representations to derive parameters of similarity normalized points (SPTS) and canonical normalized appearance (CAPP). These were trained to detect individual AUs and the PSPI metric. SPTS and CAPP solutions were then used individually and in combination to evaluate performance. With some exceptions, the individual representations performed reasonably at both AU detection and overall PSPI prediction. Combining both parameters yielded an A' value of 0.84 at predicting the PSPI index.

Hammal and Kunz (55) proposed a hybrid machine learning approach to classifying spontaneous expressions of experimental pain, based on the Transferable Belief Model. The model was based on the dynamic fusion of appearance features around the wrinkle areas (the deepening of transient facial features). Video sequences of participants responding to painful or non-painful heat stimulation were classified in a 2-alternative forced-choice paradigm, achieving a correct classification rate of 81.2%. A test of the ability of the system to correctly discriminate among pain, posed expressions of six basic emotions, and neutral expressions (an 8-alternative forced choice) achieved a correct classification rate of 84.5%. Automatic classification outperformed untrained human observers. Importantly, these findings demonstrated the feasibility of automatically differentiating pain from other emotional expressions. Unlike approaches that rely exclusively on static information from video recordings, the model incorporated temporal changes in features, thus more closely approximating the perceptual processes of human observers.

Most approaches to pain detection seek to determine only whether pain is present or absent. Hammal and Cohn (56), extended previous efforts by attempting to classify pain intensity (as opposed to presence). Using the UNBC-McMaster Shoulder Pain Expression Archive Database, they defined four pain intensity scores from the PSPI metric: none (PSPI = 0), trace (PSPI = 1), weak (PSPI = 2), and strong (PSPI > = 3). For each video frame, AAMs were first used to track and register rigid and non-rigid face motion. Based on this information, the canonical appearance of the face (CAPP) was extracted for each frame. CAPP features were then rescaled to 96 × 96 pixels and passed through a set of Log-Normal filters of 7 frequencies and 15 orientations. The extracted spatial face representation was then aligned as a vector of 9,216 features and used by four SVMs trained separately to measure the four pain intensity levels. Results showed fair-to-good classification of the intensity levels, depending on the classification accuracy metric and method of validation between training and testing data, with moderate-to-high consistency between automated measurement and the original PSPI metric. Several other researchers have described effective CVML methods for assessing pain intensity from facial expression [(45, 47, 57–62)]. In short, the data suggest that automated assessment of expressed pain intensity is feasible.

These early efforts provided an initial proof-of-concept that the occurrence of pain can be automatically measured from the face. There have since been scores of studies supporting the concept [see Werner et al. (3) for a survey of work to 2019].

### Applications in Specific Populations

Interest in evaluating pain by assessment of non-verbal expression has been driven to a significant extent by clinical concerns; in particular, the fact that large cohorts of people cannot report on their pain because of verbal communication deficits. These include infants and young children and people with neurological impairments, especially dementias. There are extensive literatures describing validated techniques for assessing pain via facial expression and other types of non-verbal behavior in neonates and young children (63) and in dementia (64). Many suffer from the same problem of burden associated with observational techniques described above; consequently, there has been a similar interest in development of automated measures for these populations.

#### Automated Assessment of Pain in Infants and Children

There have been several efforts to develop automated systems for assessing pain in infants and children (65). Most have made use of a publicly available resource, the Classification of Pain Expressions (COPE) database (66). The database consists of 200 still photographs taken of neonates during five conditions, one of which was undergoing blood sampling by lancing of the heel. In an initial study, 88% correct classification in distinguishing the response to heel lancing from pain from rest, crying, air-puff, and friction conditions was achieved with a SVM approach. In a later study, using techniques based on processing of image textures and SVM's, an Area-Under-the-Curve ROC value of 0.93 was obtained discriminating pain from non-pain conditions.

With recordings obtained from neonates undergoing heel-lancing, Zamzmi et al. (67) extracted optical flow strain measures to train a K-nearest neighbor classifier, achieving 96% correct classification distinguishing pain from no pain, as evaluated against the ground-truth of nurses' ratings on an infant pain scale incorporating assessments of facial expression, among other behaviors.

Sikka et al. (37) studied children, aged 5 to 15, during different phases of treatment for appendicitis. An automated procedure—the computer expression recognition toolbox (68)—was used to detect FACS AUs, which were then used in logistic regression to classify pain, achieving Area-Under-the-Curve values of 0.84–0.94 predicting pain.

#### Automated Assessment of Pain in Aging and Dementia

Kunz et al. (69), using FACS, showed that facial pain expressions were able to document pain among patients with dementia who could not articulate valid verbal pain ratings and that patients with dementia showed a greater pain reaction than controls. As with other applications of behavioral measurement, this knowledge has been slow to affect clinical practice because of the measurement burden problem highlighted above. This has motivated the pursuit of automated systems for evaluating pain expression in dementia.

Progress in this pursuit has recently been documented by Rezaei et al. (70). Using video recordings taken from the UNBC-McMaster Shoulder Pain Expression Archive Database and a new dataset of elderly people with and without dementia undergoing potentially painful physiotherapy maneuvers a computer vision model of fully automated detection of pain expression was developed and evaluated. The model attempted to approximate the perceptual processes of human observers, who take into account temporal changes in expression by pairing target frames and reference frames. The best performing models, when evaluated against a pain/no pain decision based on the PSPI metric, yielded Area Under the Curve values of 0.86, and 0.85 for per-frame detection of people with dementia and those without, respectively. This supports the feasibility of automatically detecting pain-related facial actions in this verbal-communication-impaired population and is all-the-more remarkable when considering the subtlety of the actions evaluated and the presence of perturbing conditions, such as body motion out of plane and variations in lighting.

### Automatic Detection of Self-Reported Pain

The bulk of this work has focused on modeling pain as represented in facial expression. More recently, however, some researchers have attempted to model other pain parameters, including sufferers' self-reports. To date, four studies have investigated automatic assessment of self-reports of pain, using video from the UNBC-McMaster Shoulder Pain Expression Archive Database. Lopez-Martinez et al. (45) proposed a two-step learning approach to estimate pain intensity as self-reported on a VAS. The approach began with a Recurrent Neural Network to automatically estimate PSPI scores at the level of individual video frames. The estimated scores were then fed into personalized Hidden Conditional Random Fields, used to estimate the self-reported VAS pain scores at the sequence level. To account for individual differences in facial expressiveness, an individual facial expressiveness score (the ratio of an independent observer's pain intensity rating) to the VAS was introduced.

A limitation of the foregoing technique is that it required retraining on previously acquired VAS ratings and thus could not generalize to previously unseen participants. To overcome this limitation, Liu et al. (59) employed another set of predefined personalized features (i.e., age, gender, complexion) to automatically estimate self-reported VAS ratings. The authors combined facial shape with these features to train an end-to-end combination of Neural Network and Gaussian Regression model (named DeepFaceLIFT), for VAS pain intensity measurement from video.

Szczapa et al. (61), proposed a video-based measurement of pain intensity scores using the dynamics of facial movement. Gram matrices formulation was used for facial point trajectory representations on the Riemannian manifold of symmetric positive semi-definite matrices of fixed rank. Curve fitting and temporal alignment were then used to smooth the extracted trajectories. A Support Vector Regression model was then trained to encode the extracted trajectories into ten pain intensity levels consistent with the VAS pain intensity measurement.

Erekat et al. (57) proposed a spatio-temporal Convolutional Neural Network–Recurrent Neural Network (CNN-RNN) model for automatic measurement of self-reported pain and observed pain intensity, respectively. The authors proposed a new loss function that explored the added value of combining different self-reported pain scales in order to improve the reliability of pain intensity assessment. Using an automatic spatio-temporal architecture, their results showed that enhancing the consistency between different self-reported pain intensity scores enhances self-reported pain estimation.

## Limitations, Constraints, and Perils of Automated Assessment of Pain

Progress toward automated analysis of pain in the past decade has been steady; nevertheless, the field is still in early development. It is a prudent time to consider some of the limitations of the approaches developed so far and problems that further studies will have to acknowledge or confront.

### Alternatives for Ground Truth

Most efforts for automatic assessment of facial expression of pain have focused on frame-level pain intensity measurement such as the FACS-based PSPI metric. The emphasis on frame level scores, from static images or a subset of images, is consistent with approaches to objective AU detection more generally.

An alternative, simpler, approach to assessing facial expression in pain is the judgment study. Using this technique, raters, who may be naïve or could have varying levels of sophistication (e.g., being trained to recognize FACS AUs or having clinical experience with pain), view recordings of subjects who may be in pain and evaluate how much pain they appear to be in by using some kind of rating scale. The number of raters can be adjusted to meet a target reliability criterion for averaged ratings (e.g., intraclass correlation ≥ 0.80) (71). The obtained aggregate scores can then be used as the ground truth of pain intensity score. The judgment study approach is more suitable to evaluating pain intensity at the sequence level because frame-level evaluation is beyond human resolving capacity. It is possible, however, that paradigms that combine slow-motion replay with use of a dial/joystick manipulandum could capture temporal changes in pain action with sufficient reliability and sensitivity to render meaningful measurement. Considering their greater simplicity and reduced burden, it is somewhat surprising that judgment study approaches have not been employed to a greater extent in studies of automated pain assessment. Indeed, because they are based on a holistic analysis that does not assume independence of an expression's component actions and probably represent human perceptual processing more realistically, they likely have advantages over measurement of specific facial actions.

### Generalizability

With few exceptions [e.g., (37)], previous efforts in automatic assessment of pain have focused on a single type of pain [shoulder pain, controlled heat; (3, 72)]. Pain comes in a variety of types, differing by modality (heat, electric, chemical), site, nature (clinical vs. artificial), and history (acute vs. chronic) that may produce different behavioral responses both within and across modalities. Given the variety of pain experiences, a variety of procedures, both experimental and observational, participants, and sensors are needed (72). The models and solutions that have shown promise for automatic detection are based on limited sampling. There is considerable evidence from direct facial measurement studies that facial expressions of pain involve a common core of actions (23, 52), but recent findings indicate that those actions come in different clusters (73, 74). This points to a need to collect further databases that sample a broader range of pain types as a way to assess the generality and generalizability of extant and novel models and solutions.

An important related need is to test approaches individually and in head-to-head comparisons across multiple databases. No studies have explicitly trained and tested classifiers on different databases in order to evaluate generalizability of automatic pain assessment across databases. Unless generalizability between separate databases is examined, it remains unknown whether methods developed in one database would be valid in others.

Care needs to be taken to address other issues of generalizability as well. Three crucial dimensions that need to be taken into account are “race,” gender, and ethnicity (75). There is an ample literature showing that, apart from facial actions, skin color coding for race has a significant effect on how pain in others is judged (76, 77), and equally abundant literatures showing that race and sex affect pain treatment and outcomes (78, 79). With the exception of the non-publicly available database collected by Sikka et al. (37) demographic information is incomplete or lacking in many instances. In future research, it will be important to systematically collect participants' demographic information to investigate the variance/invariance of pain experience and measurement in order to provide a more comprehensive assessment of pain occurrence and intensity.

### Bias

There is recent evidence that algorithms arising from deep-learning approaches to processing the face perform differently as a function of race and sex (for example at facial recognition), sometimes to a considerable degree (80). Likely a consequence of the fact that the datasets used for training largely sample unrepresentatively; i.e., from young, light-skinned, male populations, increasing awareness of the existence and implications of algorithms that are biased raises serious concerns about issues of fairness. The issue has become of sufficient general concern to lead to calls to ban certain applications of artificial intelligence, including work on mental health diagnosis and detection of deception (81).

That the issue of biased behavior of algorithms likely applies to detection of pain was demonstrated by Taati et al. (82), who compared the performance of currently available facial landmark and facial action unit detection algorithms on a dataset consisting of facial expressions showing various degrees of pain in a population of older people with dementia and older people living independently. Ground truth was landmark identification and facial action unit coding by human experts. Performance of the pre-trained algorithms at landmark detection was significantly better for independent-living seniors than for those with dementia. Retraining the algorithms with representative examples of faces of independent-living and seniors with dementia was able to improve performance significantly. With respect to detecting facial action units by available pre-trained algorithms, there was no difference between independent-living seniors and those with dementia, possibly because the algorithms performed poorly in general. The results emphasize the importance of sampling broadly and representatively with respect to subject group and type of pain and highlight the need for extreme caution against overgeneralizing about what the results of automated analysis show, particularly as the field moves inexorably toward implementation in clinical settings.

### Fully Automatic Multimodal Pain Assessment

By far, most efforts at automatic analysis of pain have focused on the face. However, pain produces multiple behavioral responses (e.g., facial expressions, head and body movements, vocalizations) both within and across modalities. Various observational systems have been developed for quantifying other behaviors indicative of pain. Some are generic and can be applied or adapted to different types of pain [e.g., (83, 84)]; others have been developed for specific purposes or populations [e.g., the Pain Assessment Checklist for Seniors with Limited Ability to Communicate; (85); the Pain Assessment in Advanced Dementia scale; (86)]. In physical medicine and rehabilitation, body language is an important behavioral index of pain in patients with moderate to severe cognitive impairments, and those who have difficulty communicating verbally (87). Non-verbal (e.g., screaming, sounds of distress) and verbal (e.g., “ouch,” “owie”) pain vocalizations have proven clinically useful for pain detection in young children and others with limited linguistic abilities (88). There is strong likelihood that automatic analysis of acoustic characteristics of vocal expression can contribute to pain detection and understanding.

There is a nascent literature that has begun to apply the methods of machine learning to these other behavioral indicators of pain [e.g., (4)]. Efforts are needed to extend CVML technologies sensing beyond facial expression to include body and head movement, physiological measures, speech, and paralinguistic communication related to pain experience.

Automatic multimodal measurement affords potentially rich sets of behavioral features to include in automatic measurement of the occurrence and intensity of pain. Newer databases that include multimodal measures, such as EmoPain and BioVid make this development possible. Efforts in this direction will enable the objective measurement and monitoring of pain intensity in clinical, family, and work environments (2).

### Links to Concepts of Expression in Pain

For all its technological sophistication, there is a kind of dustbowl empiricism about the corpus of work on automated analysis of pain. Although it builds on prior knowledge and findings–in particular the literature applying fine-grained behavioral analysis to the characterization of expression in pain–for the most part it has not addressed conceptual issues related to its meaning. Behavioral studies suggest that there is considerable complexity in the facial behavior that accompanies pain. Kunz and Lautenbacher (73), for example, provide evidence that the actions that most consistently relate to pain in the literature occur in separable clusters. This is an issue that has not been addressed in the _automated_ assessment literature. Moreover, there is good reason to believe that not all the expression that happens in pain is about pain. For example, the action of zygomaticus major (AU 12 in FACS), which is also the principal movement in a smile, is sometimes found to accompany pain, both in the behavioral literature (89) and the automatic analysis literature. Structural and functional analyses of this action suggest that, although it often does accompany pain, it is likely marking a different process (41, 90). CVML models to date do not seem to have recognized this distinction yet may have analytic potential to advance its understanding. Similarly, there is evidence that different components of the behaviors that correlate with pain are encoding different dimensions of the experience. Kunz et al. (91) found that actions involving movement around the eyes related most closely to sensory features of pain, while movements of the brows and upper lip related most closely to affective features. CVML studies have not addressed such issues to date but could be important in advancing our understanding of them.

### Commercial and Other Applications

Commercial tools for pain assessment informed by the existing literature on automated assessment have already been developed and marketed and there is every reason to believe that this trend will continue. For example, Painchek (www.painchek.com) is a smartphone app-based device that combines a facial expression assessment component with input from five other domains (voice, movement, behavior, activity, body) to yield a pain score for application in geriatric and pediatric settings (92). It goes without saying that the development and marketing of tools for clinical assessment should be based on knowledge about automated assessment that is grounded in the empirical literature, consistent with the best-established technological solutions, has been subjected to rigorous validation procedures, and informed by understanding of issues of bias raised above. Importantly, commercial applications must be cognizant of the risks attendant on oversimplified interpretation of the meaning of a pain score derived from automated analysis of the face. An oft-stated rationale for focusing on facial and other behavioral indicators of pain is to improve pain management by improving pain detection. There is a substantial literature, however, showing that observers underestimate behavioral evidence of pain (93). This underestimation bias is paradoxical given that significant proportions of subjects in empirical studies show no behavioral evidence of pain (94). Facial expressions of pain have been characterized as a “late signaling system” (95), which implies that, if facial evidence of pain is present, it is likely very significant and needs to be taken seriously. Conversely, if it is not present, the possibility of its significance should not be discounted, a risk that is present with oversimplified interpretation of pain scores, however rendered.

A related concern arises from what appears to be widespread interest in the idea of pain simulation and empirical work implying that genuine pain can be distinguished from dissimulated pain. The idea lends itself to considerations that there may be forensic applications of automated assessment technology. It is true that perceptual (52), behavioral (96), and now automated assessment studies (36) have shown evidence that facial expressions during genuine and simulated pain have certain identifiable differences; however, the differences that have been documented have occurred under highly artificial conditions and appear, for the most part, to be small. Foreseeable application of forensic products based on automatic analysis appear open to abuse and unlikely to be probative.

## Whither Automated Assessment of Pain?

That automated analysis of pain may be feasible has been demonstrated in the proof-of-concept studies reviewed above. The numerous studies that make up the corpus of the field since then have mainly added to the field by exploring alternative artificial intelligence systems. Ultimately, the value of this work is most likely to be realized in basic science and clinical research. In particular, the prospect of a form of assessment that can automatically yield reliable, valid and continuous information about how and when people (and animals) are expressing pain holds promise to enable detailed studies of pain modulation that are prohibitively difficult to perform with human observers who are subject to inherent limitations in their ability to resolve changes in behavior that sometimes occur in milliseconds, fatigue, and error. This could include evaluations of the time-course of pain reducing or augmenting influences but it could also extend to studies of how intrapersonal variables and the interpersonal, social, and environmental context influence pain over momentary differences in time. There is evidence from extant studies that automated detection techniques can give insight into momentary changes at or near the level of a frame of video [(51); see Figure 1]. In principle, valid measurement at that level of sensitivity could yield important information about dose-response relationships in evaluations of analgesic medications. A system that combined automated detection of pain with detection of other affective states (e.g., anger) and also permitted time-series analysis could facilitate greater understanding of the interplay of the states. To date, no attention has been applied to how automatic pain detection may vary between men and women, people of different racial and ethnic backgrounds, or context, to name just a few factors. Of particular interest in would be studies of pain expression in interactions in health-care settings or in families.

The work performed to date for automated pain measurement has been interesting, progress has been rapid and has generated the kind of buzz commonly associated with new technologies. But numerous current controversies over unforeseen consequences about how these new algorithms have been developed (for example, errors that have been “baked in” to the data on which facial recognition systems were trained, leading to wrongful arrest), or how they work highlight the need to proceed cautiously, mindful that “move fast and break things” is not a slogan that augurs well for the careful and safe development of a tool to advance understanding of pain in particular and other health related applications in general. The existing approaches are built on a very limited sample of participants, pain types, annotation procedures, conditions of observation, ages, “racial”/ethnic categories, and regions of the world. Careful expansion of audiovisual pain databases that sample more broadly and representatively across these dimensions will be necessary to establish confidence in the quality and meaning of the measurement obtained and to manage foreseeable and unforeseeable perils of using this technology to improve patients' outcomes. Particular concern arises around the prospect of developing and commercializing technologies geared to clinical, medico-legal, and forensic applications, especially around the idea of proprietary knowledge. Practical applications of automatic pain assessment need to be based on rigorous science that meets standards of professional peer review and public accountability, including verification that the CVML processes on which they are based validly produce assessments that are consistent with the claims being made of them.

## Author Contributions

KP and ZH contributed to conceptualization and writing of this paper. Both authors contributed to the article and approved the submitted version.

## Funding

This work was supported in part by the National Institutes of Health under Award Number R01NR018451. The content is solely the responsibility of the authors and does not necessarily represent the official views of the National Institutes of Health.

## Conflict of Interest

The authors declare that the research was conducted in the absence of any commercial or financial relationships that could be construed as a potential conflict of interest.

## Publisher's Note

All claims expressed in this article are solely those of the authors and do not necessarily represent those of their affiliated organizations, or those of the publisher, the editors and the reviewers. Any product that may be evaluated in this article, or claim that may be made by its manufacturer, is not guaranteed or endorsed by the publisher.
