# Corrigendum: Computer Mediated Automatic Detection of Pain-Related Behavior: Prospect, Progress, Perils

**DOI:** 10.3389/fpain.2022.849950

**Published:** 2022-02-25

**Authors:** Kenneth M. Prkachin, Zakia Hammal

**Affiliations:** ^1^Department of Psychology, University of Northern British Columbia, Prince George, BC, Canada; ^2^The Robotics Institute, Carnegie Mellon University, Pittsburgh, PA, United States

**Keywords:** pain, measurement, facial expression, automation, assessment

In the original article, the subheading **Assessment Based on Fine-Grained Facial Observation** should have been deleted. The correct subheading is: **Pain Assessment Based on Fine-Grained Facial Observation**.

In the original article, the subsection titled **Limitations of Physiological Assessment of Pain** appeared in an illogical place, immediately after a new section and topic, **Pain Assessment Based on Fine-Grained Facial Observation**, is introduced. The correct location of the subsection is directly after the section titled **Physiological Assessment of Pain**.

In the original article, the authors were inconsistent in the labeling used to refer to the UNBC-McMaster database. Because of how it was designated in the original article describing it, how central it is to the field, and so that researchers in the field can cite and track work based on it accurately, the term “UNBC-McMaster” has been corrected to be referred to uniformly as the “UNBC-McMaster Shoulder Pain Expression Archive Database.”

In several places in the original article, where the UNBC-McMaster Shoulder Pain Expression Archive Database is described, the sentence construction was awkward for lack of the preceding definite article, “the”. This has been corrected on pages 6, 7, and 8.

In the original article, there was an error in the **Funding** statement. By official US National Institutes of Health policy, the **Author Disclaimer** statement must follow the funding acknowledgement. The **Author Disclaimer** has been moved to the **Funding** statement.

In the original article, acknowledgment of support was inaccurately not contained in an **Acknowledgments** statement. The acknowledgment of Canadian Institutes of Health Research funding gives the correct grant information and does not require the disclaimer.

The corrected **Funding** statement and **Acknowledgments** statement appear below.

## Funding

This work was supported in part by the National Institutes of Health under Award Number R01NR018451. The content is solely the responsibility of the authors and does not necessarily represent the official views of the National Institutes of Health.

## Publisher's Note

All claims expressed in this article are solely those of the authors and do not necessarily represent those of their affiliated organizations, or those of the publisher, the editors and the reviewers. Any product that may be evaluated in this article, or claim that may be made by its manufacturer, is not guaranteed or endorsed by the publisher.

